# Outpatient psychotherapeutic treatment of gambling disorder — lessons learned from Bavaria

**DOI:** 10.3389/fpsyt.2026.1760669

**Published:** 2026-02-18

**Authors:** Bianca Pitzschel, Rebekka Redel, Martin Tauscher, Gabriele Koller, Elena Gomes de Matos, Eva Hoch, Larissa Schwarzkopf

**Affiliations:** 1 IFT Institut für Therapieforschung, Centre for Mental Health and Addiction Research, Munich, Germany; 2 Department of Psychiatry and Psychotherapy, LMU University Hospital, LMU Munich, Munich, Germany; 3 Bavarian Association of Statutory Health Insurance Physicians (BASHIP), Munich, Germany; 4 Department of Clinical Psychology and Psychotherapy, Charlotte Fresenius University, Munich, Germany; 5 Institute for Medical Information Processing, Biometry, and Epidemiology – IBE, LMU Munich, Munich, Germany; 6 Pettenkofer School of Public Health, Munich, Germany

**Keywords:** characterization help-seeking gamblers, comorbidities, gambling disorder, medical usage, outpatient psychotherapy, outpatient treatment, problematic gambling, psychotherapist care

## Abstract

**Background:**

Psychotherapeutic interventions are an effective treatment for gambling disorder (GD), yet little is known about their uptake in routine care settings. Using data from the Bavarian Association of Statutory Health Insurance (SHI) Physicians, this study estimates the proportion of patients with disordered gambling behavior receiving psychotherapy, describes their diagnostic characteristics, and outlines treatment patterns.

**Methods:**

Adults insured by SHI in Bavaria with a confirmed GD diagnosis (ICD-10: F63.0 G) according to the M2Q criterion (i.e., diagnosis in ≥ 2 out of 4 quarters) who received outpatient care between January 2021 and March 2022 were included. Patients were divided into three groups, comprised of patients with (1) at least one billable encounter with a psychotherapist (PT), (2) at least one billable encounter with a neurologist or psychiatrist but none with a PT, and (3) no billable encounters with either. Groups were compared by age, sex, diagnosed comorbid mental disorders, and service utilization. Key elements of psychotherapy were also analyzed.

**Results:**

Of 3,154 patients with disordered gambling behavior, 589 (18.6%) received psychotherapy. This group was younger (*M* = 39.7 years, *SD* = 12.8) than group 2 (*M* = 44.9 years, *SD* = 13.8) and group 3 (*M* = 43.8 years, *SD* = 14.1) and included a higher proportion of women than group 3 (18.5% vs. 12.4%). Comorbid depression, anxiety, and adjustment disorders were more frequent among patients in psychotherapy. Compared to group 3, they received services from internists, organ-specific specialists, and technical specialists more often. Compared to group 2, they more often used psychiatrists’ services. Psychotherapy was most commonly individual cognitive behavioral therapy (CBT) and lasted 352 days on average, with about 18 sessions per patient.

**Conclusion:**

Patients with disordered gambling behavior (according to ICD-10 criteria for F63.0) who receive psychotherapy often have additional mental disorders and higher overall care needs. This highlights the importance of integrated treatment approaches. The frequent use of CBT aligns with evidence-based practice and suggests potentially favorable therapeutic outcomes.

## 1 Introduction

Gambling disorder (GD) — classified as a behavioral addiction in both the International Statistical Classification of Diseases 11th Revision (ICD-11: “Gambling disorder”), and the Diagnostic and Statistical Manual of Mental Disorders, Fifth Edition (DSM-5: “Gambling disorder”) (1, 2) — is characterized by persistent or recurrent online or offline gambling, marked by loss of control, prioritizing gambling over interests and daily activities, continuing despite harm, and causing significant distress or impairment over at least 12 months (1, 2). GD is linked to multiple detrimental consequences such as financial hardship, strained relationships, productivity loss, psychological distress and elevated health risks (3). In addition to these gambling-associated impairments, individuals with disordered gambling behavior often present comorbid mental disorders such as depression, anxiety disorders, substance use disorders, post-traumatic stress disorder or attention-deficit hyperactivity disorder (4–7), which presumably reinforce the detrimental “impact” of gambling-associated harm (8).

Due to the considerable burden associated with GD, treatment options play an important role in helping individuals with disordered gambling behavior. In absence of an approved effective pharmacotherapy for GD (9), psychotherapy is an acknowledged promising treatment approach for GD (10, 11), which has already proven its effectiveness in treating common psychiatric comorbidities of GD such as depression, anxiety disorders or post-traumatic stress disorder (12–15).

According to meta-analytical evidence, cognitive behavioral therapy (CBT) in particular has a strong mitigating effect on gambling severity (10, 16–18), a moderate effect on gambling frequency (16–18), and a weak effect on gambling intensity (16, 17). Evidence on the effectiveness of motivational interviewing is mixed, with two systematic reviews finding no effects on symptom severity (10, 18), one reporting small effects on gambling frequency (18) as well as moderate effects on financial losses (18), and another review indicating significant effects of unclear sizes on gambling frequency and financial losses (19). A meta-analysis from 2022 demonstrated the efficacy of exposure therapy in diminishing craving, gambling severity, and gambling frequency (20). In addition to studies examining the efficacy of different therapeutic approaches, there is evidence on the effects of various kinds of therapeutic interventions and their mode of delivery. Two meta-analyses demonstrated a small beneficial effect of brief interventions on gambling behavior (21, 22) and guided online interventions or in-person psychotherapy showed better effects than remote or self-guided psychotherapy interventions (10, 23, 24).

In Europe, professional treatment options for individuals with gambling-related problems often include psychotherapy or psychotherapeutic interventions in inpatient or outpatient settings, with country-dependent differences in providers and reimbursement (25). For example, in the UK, the National Health Service provides gambling treatment in a clinical setting and charities and community organizations offer gambling treatment in community and residential settings in parallel — making concomitant treatment possible — and there are regional differences and variations in the qualifications and medical disciplines of staff, as well as in treatment approaches (26). While the National Health Service employs clinical experts like general practitioners or psychiatrists, mental health professionals (registered or not), and peer-support experts, the occupations and qualifications of the staff of non-profit sector institutions vary, as do treatment options. Treatments range from psychologist-led or registered therapist-led CBT and pharmacological interventions (mostly National Health Service) to motivational interviewing (most National Health Service and non-profit institution services) to brief interventions and structured psychosocial interventions (non-profit institutions) to other interventions, such as group or family therapy or general counselling by certain practitioners.

In Germany, professional treatment for individuals with disordered gambling behavior comprises counseling from outpatient addiction care facilities; inpatient and outpatient rehabilitation programs (offered at inpatient clinics and outpatient clinics, respectively); and outpatient therapy with a licensed (medical or psychological) psychotherapist (PT) (27). Compared to inpatient rehabilitation and outpatient psychotherapy, outpatient addiction care facilities provide a low-threshold entry point into to the care system without waiting times (ideally), need for registrations or application process at the cost-bearer. Particularly for individuals with complex clinical and psycho-social profiles, outpatient psychotherapy offers the advantage of patient-individually tailored treatment plans. Thus, integrative addressing of GD and comorbid mental disorders is rather possible in outpatient psychotherapy than in inpatient rehabilitation programs that in general have a distinct focus on one distinct primary diagnosis (GD OR the comorbidity). All these services are nearly free of charge, as most costs are typically covered by pension insurance, health insurance, or municipalities or federal states (27).

Availability of these offers varies geographically and it is presumable that — just as studies in several other countries showed (28–30) — only a small fraction of individuals with gambling-related problems in Germany seek professional help. This notion is supported by a Bavaria-based survey conducted in 2009 which found that roughly 150 to 500 individuals with gambling-related issues received psychotherapeutic treatment (mainly CBT) that year (31).

So far it remains unclear whether these treatment rates are still representative of patients with gambling-related issues in Bavaria, which other outpatient practitioners treat patients with disordered gambling behavior, and whether patients with disordered gambling behavior in psychotherapeutic treatment comprise a distinct subgroup of patients with GD.

Therefore, the objectives of this study were:

- to determine the share of patients with disordered gambling behavior who receive outpatient psychotherapeutic treatment among all patients with disordered gambling behavior receiving outpatient physician treatment.
- to compare patients with disordered gambling behavior with and without psychotherapeutic treatment to assess any differences in billable encounters with practitioners in various medical disciplines.
- to compare the demographic and comorbidity-related characteristics of patients with disordered gambling behavior with and without psychotherapeutic treatment.
- to describe the type of psychotherapeutic treatment received.

## 2 Materials and methods

### 2.1 Participants and data collection

#### 2.1.1 Data source

Analyses were performed using insurance claims data provided by the Bavarian Association of Statutory Health Insurance Physicians (German: “Kassenärztliche Vereinigung Bayerns”) covering the period from 01 January 2021 to 31 December 2023. These data reflect a full census of all outpatient services (including psychotherapeutic treatment) billed to all Statutory Health Insurances in the State of Bavaria. Each instance of a billing code was considered to represent the occurrence of a billable encounter with an outpatient health care professional. As all encounters in the data set were billed, we refer to these simply as encounters in this study. Among adult (≥18 years) residents of Bavaria, roughly 85% of 5.4 million males and roughly 90% of 5.5 million females were insured by Statutory Health Insurance (SHI) in 2021 (32, 33). Statutory Health Insurance is financed via income-based, risk-independent premiums and provides access to a broad range of inpatient and outpatient services with free or low-cost access at the point of service (34).

In Germany outpatient psychotherapy is a regular part of the bundle of services covered by the statutory health insurance. Thus, several components of psychotherapeutic treatment can be accessed without the patients’ copayment: Here, consultation hours (up to 6 sessions per 4 quarters, 25 minutes each) and probatory sessions (up to 4 sessions per 4 quarters, 50 minutes each) before start of the actual therapy serve for diagnosis and assessment of treatment needs. The subsequent therapy covers short-term (up to 24 sessions, 50 minutes each) and long-term courses (up to 60 sessions, 50 minutes each), acute outpatient treatment (up to 24 sessions, 25 minutes each) as well as (psychotherapeutic) conversations (up to 150 minutes per quarter of a year). Each service has a maximum quota that can be used if necessary. Short-term courses and long-term courses are subject to approval from the SHI, whereas acute outpatient treatment only requires notification of the patient’s SHI. Provision of psychotherapeutic consultation hours, probatory sessions or (psychotherapeutic) conversation is not linked to any further requirements.

Due to data protection standards of the Association of Statutory Health Insurance Physicians of Bavaria, all data were provided in pivot tables (i.e., as aggregate data). Therefore, it was not possible to analyze the data of individual patients or examine individual-level parameter distributions. Consequently, we were unable to account for heterogeneity in patient-individual covariate profiles through adjusted regression. This necessitates that the observed differences are interpreted sensitively whilst taking into consideration group-specific covariate structures.

#### 2.1.2 Sample selection

Diagnoses in the Statutory Health Insurance system rely on the German Modification of the ICD-10 (ICD-10 GM) (35) and are reported per quarter of the year. We included all patients ≥ 18 years old with disordered gambling behavior — diagnosed based on ICD-10 criteria for “Pathological Gambling” (F63.0) — between Q1–2021 and Q1–2022 who received that diagnosis again in at least one of the three consecutive quarters. This so-called M2Q criterion (i.e., presence of the diagnosis in at minimum two quarters in a four-quarter period) is widely used in claims data analyses to avoid false positives in classification (36, 37). The timing of the first of these diagnoses determined the patient’s index quarter – which must not be misinterpreted as the quarter in which the initial diagnosis was given. Each patient was followed for eight quarters, with observation periods spanning from the period between Q1–2021 and Q4–2022 to the period between Q1–2022 and Q4 2023. We excluded patients with at least one confirmed diagnosis of Parkinson’s disease (ICD-10 code G20 G, G21 G, or G22 G) during the assessment period because disordered gambling behavior is an acknowledged side effect of certain anti-Parkinson drugs (38, 39).

All patients meeting the inclusion criteria were subsequently divided into three separate groups: group 1 contained all patients with ≥ 1 encounter with a (medical or psychological) PT (~ billing of at least one psychotherapeutic service by any qualified practitioner); group 2 contained all patients with ≥ 1 encounter with a psychiatrist or a neurologist (providing mainly pharmacotherapy and enhanced diagnostics), but without any billed psychotherapeutic services by any practitioner; and group 3 contained all patients with neither PT nor neurologist or psychiatrist encounters (~ no billing of any psychotherapeutic or neurological or psychiatric services by any practitioner).

### 2.2 Outcomes

To answer research question 1, we determined what share of the total sample belonged to group 1.

To answer research question 2, we examined the encounters with practitioners in various medical disciplines (yes/no) as well as the average number of annual encounters per patient. The following medical discipline categories were considered:

- general practitioners;
- internists;
- organ-specific specialists;
- specialists in technical, laboratory, and pathology disciplines;
- psychiatrists and neurologists;
- PTs; and
- specialists from other disciplines.

In this study, we refer to specialists in category (d) as technical specialists. Practitioners with additional training as PTs, as well as specialists in psychiatry and psychotherapy and specialists in psychosomatic medicine and psychotherapy, were categorized as medical PTs if they billed for a psychotherapeutic service during the observation period. Category (e) was subdivided into neuropsychiatrists, neurologists, and psychiatrists. Category (f) was subdivided into medical PTs, psychological PTs (German: “psychologische Psychotherapeuten”) specializing in (cognitive) behavioral therapy, and psychological PTs specializing in depth psychotherapy or psychoanalysis (German: “Tiefenpsychologisch fundierte Psychotherapie”, “analytische Psychotherapie”). The mean number of encounters in the two-year period was reported. Due to the similarity of values for the average number of annual encounters, we report the average number of both years instead of separate means for each year.

To answer question 3, we defined the presence of comorbid mental disorders as ≥ 1 confirmed diagnosis during the observation period. Based on the existing literature (4, 6), the prevalence of alcohol use disorder (F10.1, F10.2); cannabis use disorder (F12.1, F12.2); nicotine dependence (F17.2); bipolar disorder (F31); depression (F32, F33); anxiety disorders (F40.0, F40.1, F41.0, F41.1); obsessive-compulsive disorder (F42); post-traumatic stress disorder (PTSD) (F43.1); adjustment disorder (F43.2); personality disorders (F60, F61); and attention deficit hyperactivity disorder (F90.0) were calculated. Furthermore, distributions of age and sex were examined.

To answer question 4, we calculated the duration of psychotherapeutic care (in days) and the number of encounters with PTs (per patient) in group 1 during the two-year observation period. Additionally, we delineated the use of distinct psychotherapeutic services using service-specific billing codes, except when small sample sizes (cell count <20) prevented the analysis. All pre-selected billing codes are listed in **Supplementary Table 1**.

### 2.3 Analysis

We calculated the means of all continuous variables (age, number of encounters). Frequencies of all categorical variables (share of patients what were female, prevalence of psychiatric comorbidities, use of distinct physician or psychotherapeutic services) were calculated as percentages. Due to the aggregate data structure, group differences could only be assessed via comparisons of 95% confidence intervals (CIs), with non-overlapping CIs indicating a significant difference at an alpha-level of 0.05.

## 3 Results

Out of all 9,439,845 SHI-insured adult residents of Bavaria, 3,154 were diagnosed with GD (M2Q criterion, ~ 0.03%) during the observation period, which translates to a prevalence of 33.4 per 100,000 SHI-insured persons. Of those, 18.7% received treatment from a PT (group 1; *n* = 589); 36.6% received care from a neurologist/psychiatrist but not a PT (group 2; *n* = 1,153); and 44.8% received care from practitioners in other disciplines, not PTs or neurologists/psychiatrists (group 3; *n* = 1,412).

### 3.1 Encounters with outpatient practitioners

Virtually all SHI-insured patients with disordered gambling behavior had encounters with general practitioners (see **Table 1**). More than two thirds of SHI-insured patients with disordered gambling behavior in group 1 had encounters to neurologists/psychiatrists in addition to their encounters with PTs. Of the subcategories of neuropsychiatric care, encounters with psychiatrists were more likely in group 1 (58.0%, 95% CI [53.2%-62.8%]) than in group 2 (46.7%, 95% CI [43.8%-49.5%]), while the share of patients using services in all other subcategories was similar. Encounters with internists were more likely in group 1 (43.8%, 95% CI [39.8%-47.8%]) than in group 3 (30.0%, 95% CI [27.6.%-32.4%]), and so were encounters with organ-specific specialists (group 1: 84.0%, 95% CI [81.1%-87.0%]; group 3: 75.4%, 95% CI [73.2.%-77.7%]) as well as encounters with technical specialists (group 1: 87.8%, [85.1%-90.4%]; group 3: 81.7%, 95% CI [79.6.%-83.7%]).

**Table 1:** Encounters with distinct practitioners (two-years observation period).

Distinct practitioners	Group 1 (N = 589) >= 1 encounter with psychotherapist	Group 2 (N = 1,153) >= 1 encounter with neurologist/psychiatrist, no psychotherapist	Group 3 (N = 1,412) No encounter with psychotherapist, neurologist or psychiatrist
General practitioners	99.8%	99.0%	100.0%
	[99.5% - 100.0%]	[98.5% - 99.6%]	[n/a]
Internists	43.8%	44.6%	30.0%
	[39.8% - 47.8%]	[41.7% - 47.4%]	[27.6% - 32.4%]
Organ-specific specialists (without internists)	84.0%	79.8%	75.4%
	[81.1% - 87.0%]	[77.5% - 82.1%]	[73.2% - 77.7%]
Specialists in technical, laboratory, and pathology disciplines	87.8%	86.8%	81.7%
	[85.1% - 90.4%]	[84.9% - 88.8%]	[79.6% - 83.7%]
Specialists from other disciplines	34.1%	32.7%	29.6%
	[30.3% - 38.0%]	[30.0% - 35.4%]	[27.2% - 32.0%]
Psychiatrists and neurologists	69.9%	100.0%	due to the group definitions, these fields are empty	
	[66.3% - 73.7%]	[n/a]		
Neurology & psychiatry combined	54.1%	50.2%		
	[49,3% - 58.9%]	[47.3% - 53.1%]		
Neurologists	30.1%	28.2%		
	[25.7% - 34.5%]	[25.6% - 30.8%]		
Psychiatrists	58.0%	46.7%		
	[53.2% - 62.8%]	[43.8% - 49.5%]		
Psychotherapists (PT)	96.1%*			
	[94.5% - 97.7%]			
Medical psychotherapist	28.8%			
	[25.1% - 32.5%]			
Psychological psychotherapist (cognitive) behavioral therapy	62.2%			
	[58.2% - 66.2%]			
Psychological psychotherapist psychoanalytic or depth therapy	20.5%			
	[17.2% - 23.8%]			

*Due to the low number of persons with contact to a psychotherapist or psychiatrist for adolescents and children those were exclude so they are missing in the percentage.

Due to multiple specializations by one practitioner the individual values could not be added up to 100%.

Among patients who received treatment, the average number of encounters per year for each medical discipline category was similar across all three groups (see **Table 2**). General practitioners were by far the most frequently seen practitioners, with the average number of encounters per year ranging from 9.6 to 10.9 across all groups. In group 1, neurologists and psychiatrists were the second most seen practitioners (*M* = 6.5 encounters per year). Organ-specific specialists were the second most seen practitioners in group 2 (*M* = 5.9 encounters per year) and group 3 (*M* = 4.5 encounters per year).

**Table 2:** Average number of annual encounters per patient (encounter with practitioner provided).

Distinct practitioners	Group 1 (N = 589) >= 1 encounter with psychotherapist	Group 2 (N = 1,153) >= 1 encounter with neurologist/psychiatrist, no psychotherapist	Group 3 (N = 1,412) No encounter with psychotherapist, neurologist or psychiatrist
General practitioner	10.5	10.9	9.6
Internists	3.5	4.0	3.6
Organ-specific specialists (without internists)	5.8	5.9	4.5
Specialists in technical, laboratory, and pathology disciplines	4.4	4.8	3.7
Specialists from other disciplines	3.0	2.5	1.5
Psychiatrists and neurologists	6.5	5.6	due to the group definitions, these fields are empty
Neurology & Psychiatry combined	4.7	4.7	
Neurologists	3.1	2.8	
Psychiatrists	6.0	5.6	
Psychotherapists (PT)	11.7		
Medical psychotherapist	9.1		
Psychological psychotherapist (cognitive) behavioral therapy	10.5		
Psychological psychotherapist psychoanalytic or depth therapy	14.2		

Patients in group 1 had 11.7 PT encounters per year on average. Encounters with PTs specializing in psychoanalytic or depth therapy tended to be more frequent (*M* = 14.2 encounters per year) than encounters with medical PTs (*M* = 9.1 encounters per year) or PTs specializing in CBT (*M* = 10.5 encounters per year).

### 3.2 Sociodemographic characteristics and frequency of mental disorders

Across all groups, females with disordered gambling behavior were in the minority (group 1: 18.5%, group 2: 15.7%, group 3: 12.4%), with a significantly higher share of females in group 1 compared to group 3 (see **Table 3**). Furthermore, patients in group 1 were significantly younger (*M* = 39.7 years) than patients in group 2 (*M* = 44.9 years) and group 3 (*M* = 43.8 years).

**Table 3:** Socio-demographics and comorbidity (N = 3.154 patients with pathological gambling).

Sociodemographics & comorbid mental disorders	Group 1 (N = 589) >= 1 encounter with psychotherapist	Group 2 (N = 1,153) >= 1 encounter with neurologist/psychiatrist, no psychotherapist	Group 3 (N = 1,412) No encounter with psychotherapist, neurologist or psychiatrist
Female (%)	18.5%	15.7%	12.4%
	[15.4% - 21.6%]	[13.6% - 17.8%]	[10.7% - 14.1%]
Age Ø	*39.7*	44.9	43.8
	*[38.7 - 40.8]*	[44.1 - 45.7]	[43.1 - 44.6]
Comorbid ICD10 mental disorder (%; 95%-CI]				
Alcohol use disorder (F10.1, F10.2)	16.8%	24.6%	20.6%
	[13.8% - 19.8%]	[22.1% - 27.1%]	[18.5% - 22.7%]
Cannabis use disorder (F12.1, F12.2)	8.0%	7.2%	4.6%
	[5.8% - 10.2%]	[5.7% - 8.7%]	[3.5% - 5.7%]
Nicotine Dependence (F17.2)	*27.5%*	36.0%	35.4%
	*[23.9% - 31.1%]*	[33.2% - 38.8%]	[32.9% - 37.9%]
Bipolar disorder (F31)	5.8%	7.2%	1.8%
	[3.9% - 7.7%]	[5.7% - 8.7%]	[1.1% - 2.5%]
Depression (F32, F33)	*85.9%*	77.0%	50.8%
	*[83.1% - 88.7%]*	[74.6% - 79.4%]	[48.2% - 53.4%]
Anxiety disorders (F40.0, F40.1, F41.0, F41.1)	*35.0%*	23.7%	10.0%
	*[31.1% - 38.8%]*	[21.2% - 26.1%]	[8.4% - 11.5%]
Obsessive-compulsive disorder (F42)	7.1%	5.3%	1.9%
	[5.1% - 9.2%]	[4.0% - 6.6%]	[1.2% - 2.6%]
Post-traumatic stress disorder (F43.1)	*13.4%*	7.3%	3.3%
	*[10.7% - 16.2%]*	[5.8% - 8.8%]	[2.3% - 4.2%]
Adjustment disorder (F43.2)	*31.9%*	21.3%	13.5%
	*[28.2% - 35.7%]*	[19.0% - 23.7%]	[11.7% - 15.3%]
Personality disorder (F60, F61)	23.9%	20.1%	10.0%
	[20.5% - 27.4%]	[17.8% - 22.4%]	[8.4% - 11.5%]
Attention deficit hyperactivity disorder (F90.0)	11.7%	9.8%	4.5%
	[9.1% - 14.3%]	[8.1% - 11.5%]	[3.4% - 5.6%]

Cursive numbers indicate a significant difference between group 1 and both other two groups (group 2 and 3).

In all three groups the most frequently diagnosed comorbid mental health disorder was depression (group 1: 85.9%, group 2: 77.0%, group 3: 50.8%) but the rankings for less frequently diagnosed comorbidities differed. In group 1, anxiety disorders (35.0%) and adjustment disorders (31.9%) ranked second and third, respectively, whereas in both group 2 and group 3 nicotine dependence ranked second (group 2: 36.0%, group 3: 35.4%) and alcohol use disorder ranked third (group 2: 24.6%, group 3: 20.6%).

In group 1, depression (85.9%), anxiety disorders (35.0%), adjustment disorders (31.9%), and PTSD (13.4%) were documented more frequently than in group 2 (depression: 77.0%, anxiety disorders: 23.7%, adjustment disorders: 21.3%, PTSD: 7.3%) and group 3 (depression: 50.8%, anxiety disorders: 10.0%, adjustment disorders: 13.5%, PTSD: 3.3%). When comparing group 1 to group 3, we found group 1 had a greater prevalence of cannabis use disorders (group 1: 8.0%, group 3: 4.6%); bipolar disorder (group 1: 5.8%, group 3: 1.8%); obsessive-compulsive disorder (group 1: 7.1%, group 3: 1.9%); personality disorders (group 1: 23.9%, group 3: 10.0%); and attention deficit hyperactivity disorder (group 1: 11.7%, group 3: 4.5%).

In contrast, nicotine dependance was diagnosed less frequently in group 1 (27.5%) than in groups 2 and 3 (group 2: 36.0%, group 3: 35.4%) and alcohol use disorders were less common in group 1 (16.8%) than in group 2 (24.6%).

### 3.3 Psychotherapeutic treatment for SHI-insured patients with disordered gambling behavior

Most SHI-insured patients with disordered gambling behavior with psychotherapeutic treatment received treatment from a psychological PT specialized in CBT (62.2%), a bit less than one third saw a medical PT (28.8%), and one fifth saw a psychological PT specialized in psychoanalytic therapy (20.5%; see **Table 1**).

The average treatment duration was roughly one year (*M* = 351 days, 95% CI [329.6-371.9]) with an average of 18 PT encounters per patient (*M* = 17.7 encounters, 95% CI [16.2-19.2]) in the two-year observation period. Assuming the encounters are approximately evenly distributed, encounters took place approximately once every three weeks.

Looking at the distinct billing codes for SHI-insured patients with disordered gambling behavior (**Table 4**), psychotherapeutic consultation hours were received by two out of every three (63.2%), individual behavioral therapy (39.0%) and probatory sessions (38.5%) were received by two out of every five, and (psychotherapeutic) conversations were received by one third of SHI-insured patients with disordered gambling behavior (34.3%). A small fraction of SHI-insured patients with disordered gambling behavior received group-based psychotherapeutic treatment (< 3%).

**Table 4:** Patients per selected billing codes (in %) and average billing frequency per patient (hole observation period).

Billing codes	Percentage (N = 589) [95%-CI]	Users’ average frequency
Psychotherapeutic Consultation-hour (billable every completed 25 minutes, maximum 6 times in 4 quarters)	63.2%	5.2
	[59.3% - 67.1%]	
Probationary session (including group treatment) (billable for 50 complete minutes, only ones a day, maximum 4 times in 4 quarters)	38.5%	2.8
	[34.6% - 42.5%]	
(Psychotherapeutic) Conversation (individual treatment) from chapter 21/22/23 (billable every 10 complete minutes, maximum 15 times per quarter of a year)	34.3%	21.1
	[30.5% - 38.1%]	
Psychotherapeutic acute treatment (billable every completed 25 Minutes, multiple billing on one day possible, maximum 24 times per case of illness)	7.0%	14.0
	[4.9% - 9.0%]	
Behavioral therapy (individual treatment) (billable for 50 completed minutes, multiple billing on one day possible, scope is determined by the application)	39.0%	15.4
	[35.1% - 43.0%]	
Depth psychological psychotherapy (individual treatment) (billable for 50 completed minutes, multiple billing on one day possible, scope is determined by the application)	18.5%	18.6
	[15.4% - 21.6%]	

CI, confidence interval. Due to the small sample size (<20) the following code categories were excluded: Psychosomatics (individual treatment), hypnosis, group psychotherapy primary care, differential diagnostic clarification/verbal intervention in psychosomatic illness (chapter 35 psychosomatic), practicing interventions, (individual treatment), analytic psychotherapy (individual treatment), behavioral/analytical/depth psychological therapy (group setting)).

Conditional on receiving a distinct service at least once, SHI-insured patients with disordered gambling behavior received, on average, 21.1 (psychotherapeutic) conversations (10 minutes each); 18.6 sessions of individual, depth psychotherapy (50 minutes each); 15.4 individual CBT sessions (50 minutes each); and 14.0 acute psychotherapeutic treatments. The average numbers of psychotherapeutic consultation hours (*M* = 5.2) and probatory sessions (*M* = 2.8) were considerably lower.

## 4 Discussion

According to our data, one in five SHI-insured patients with disordered gambling behavior who receive outpatient treatment in the German SHI system receives psychotherapeutic treatment. These patients make up a subgroup with a comparatively young age, a comparatively high share of females, and a pronounced burden of comorbid mental disorders. Psychotherapeutic treatment is mainly provided by PTs specialized in CBT, and lasts for about one year, with individual treatment being standard. The frequency of CBT treatment, which is currently the most promising (psychotherapeutic) treatment approach for GD (10), suggests outpatient psychotherapeutic care is provided according to the best scientific evidence available.

In light of the 2.3% prevalence of GD among German adults (18 to 70 years) at the national level in 2021 (40), obviously only a small fraction of people with disordered gambling behavior seek outpatient physician or PT treatment (3,154 of about 7,717,101 SHI-insured patients ages 18 to 69). Even though the diagnostic criteria of GD according to the DSM-5 (41) and that of pathological gambling according to ICD-10 (42) do not perfectly match, these results substantiate previous findings on treatment gaps which are related to a mixture of lacking problem awareness (“no perceived need”) as well as individual barriers (e. g., shame, stigma, neglect) and structural barriers (such as waiting times, application procedures; unmet perceived need) (28, 43).

We found that, for SHI-insured patients receiving outpatient treatment, psychotherapeutic treatment is less relevant. This suggests a particularly high threshold for initiating psychotherapeutic services, as already demonstrated for mental health issues in general. According to a German claims data analysis, roughly one in seven SHI-insured patients with a diagnosed mental health disorder seeks treatment from a PT (44). Furthermore, studies indicate that use of mental health services due to (substance-related) addictive disorders is even less likely than use of mental health services due to other mental health issues such as anxiety disorders or depression (45). These barriers may differ by age and gender.

### 4.1 Encounters with outpatient practitioners

One of our key findings is that literally all SHI-insured patients with disordered gambling behavior in our sample saw a general practitioner during the observation period. Thus, general practitioners could play a vital role in diagnosing GD and referring patients to gambling-specific inpatient and outpatient treatments. Keeping in mind that individuals with disordered gambling behavior might not seek the care of a general practitioner for the gambling problem itself, but rather due to associated symptoms (depressiveness, anxiety, stress) (46), sensitizing general practitioners for a potential linkage of these symptoms with GD appears highly appropriate. Furthermore, studies from other countries have demonstrated that — in addition to limited resources (47) — uncertainty about how to raise the sensitive topic of (suspected) gambling-related problems in a patient-physician interaction is a major impediment among general practitioners for treating individuals with a GD diagnosis (48, 49). One study found a lack of confidence in handling patients with disordered gambling behavior among general practitioners in primary care, which could be one reason why hardly any general practitioner participating in the survey screened their patients for GD (49). Equipping general practitioners with efficient and effective screening tools, having compact information about treatment options nearby, and providing targeted training on GD would be an essential prerequisite for enabling them to treat and manage individuals with disordered gambling behavior successfully.

### 4.2 Sociodemographic characteristics and frequency of mental disorders

Statutory Health Insurance-insured patients with disordered gambling behavior with psychotherapeutic treatment had a lower mean age and a higher share was female as compared to SHI-insured patients with disordered gambling behavior without psychotherapeutic treatment. This is in line with previous evidence that males and the elderly are less likely seek psychotherapeutic treatment (50–52), and might be explained to some extent by a more positive attitude towards psychotherapeutic concepts among females (52–54) and the fact that traditional norms of masculinity discourage males from seeking psychotherapeutic support (55). Additionally, a German experimental vignette study revealed higher stigmatization scores for male vignettes than for female ones, regardless of whether the described addiction was gambling, internet use, or alcohol use disorder (56). This external stigma might further heighten barriers to seeking help among males with disordered gambling behavior. In addition, females face a higher likelihood of developing certain mental disorders (e. g., mood and anxiety disorders) than males (57), which might further contribute to the comparatively high share of females in group 1, which is also characterized by notably high psychiatric comorbidity burden.

It is plausible that the co-occurrence of GD and other mental disorders encourage patients to seek psychotherapeutic treatment, even though the key reason for treatment initiation might not be fully disentangled. Given that “gambling” is linked to more pronounced public stigma than other mental disorders such as depression or compulsive disorder (56, 58), barriers to seeking psychotherapeutic treatment when comorbid mental disorders are present may be lower than barriers to seeking psychotherapeutic treatment due to GD alone (59). Furthermore, the negative effects of the comorbid mental disorder might be recognized more easily (4) than those of GD, as self-awareness of gambling-related problems is often low among those affected (60). Among people with disordered gambling behaviors, the prevalence of certain mental disorders is higher than the prevalence within the general population. According to Germany’s national health surveillance system, the administrative prevalence — defined as at least one confirmed outpatient diagnosis within the SHI system — of depression among adults was 16.6% in 2021 (61) and that of anxiety disorders was 7.7% (62). Within the same data set, the prevalence of PTSD across all age ranges amounted to 0.9% in 2022 and that of substance use disorders amounted to 7.5% (63). This aligns with previous findings that GD is often accompanied by other mental health issues (4, 5). It also reinforces that screening for GD by PTs is necessary, especially among patients with depressive and anxiety symptoms, which would enable integrated, individualized treatment of people who have both GD and other mental disorders. Therefore, treatment concepts for people with disordered gambling behavior that also address comorbid mental disorders need to be developed and systematically evaluated.

### 4.3 Psychotherapeutic treatment for SHI-insured patients with disordered gambling behavior

The psychotherapeutic care provided most frequently within our sample corresponds to the number of hours provided in a course of short-term therapy as dictated by German health insurance regulations. Typically, short-term therapy includes approximately 12 sessions, with up to five additional 50-minute probatory sessions dedicated to diagnostics and assessing the fit between the patient and the PT.

Within our sample, psychotherapeutic treatment is mainly individual treatment, even though a meta-analysis demonstrated that face-to-face group and individual psychotherapeutic interventions are similarly effective treatments for GD (10, 24, 64). Hence, the dominance of individual psychotherapeutic treatments might not necessarily reflect a conscious choice of a superior treatment format but may reflect practical reasons. Considering that both our findings and existing literature indicate that few individuals with disordered gambling seek outpatient psychotherapeutic treatment, group therapies might fail because of a lack of participants. According to one study, each PT who treats people affected by GD has two patients each year on average – if both people with gambling-related problems and people from the social environment of those with gambling-related problems are accounted for (31). In addition, shame and fear of stigmatization may discourage people with disordered gambling behavior from participating in cross-diagnostic group formats (e.g., for emotional skills training) (65).

### 4.4 Strengths and limitations

The findings of our study need to be interpreted with design-related limitations in mind. First, using the M2Q criterion to classify patients as having GD puts the focus on a patient population which is closely connected to the outpatient system. As our aim was to illustrate outpatient care pathways, we consider this justified, even though it disregards SHI-insured patients with disordered gambling behavior who are only loosely connected to the outpatient system (e.g., because of early referral to inpatient treatment).

Second, regarding outpatient physician and psychotherapeutic care in the SHI system, the data does not allow for conclusions about the uptake of complementary treatments and support services (e.g., rehabilitation, acute care in psychiatric wards, gambling-related counselling). Hence, receipt of psychotherapeutic treatment must not be misinterpreted as access to more comprehensive GD-related care and support.

Third, as an exploratory, observational study between and within group heterogeneity cannot be ruled out: First, considering the different age and gender distribution between groups 1 to 3, all between group comparisons remain a sensitive issue. Second the documentation relies on the ICD-10 system, which does not mirror the clinical phenotype of GD (and psychiatric comorbidity). Therefore, within group heterogeneity among patients assigned to groups 1 to 3 presumably persists. The decision to base group assignment on ≥1 encounter with a neuropsychiatrist (group 2) or a psychotherapist (group 1) supposedly reinforced this issue, as patients with different treatment intensity are collapsed. Psychotherapeutic treatment is more effective at less advanced stages of GD (66), thus it cannot be ruled out that non-access to PT care is driven by non-eligibility of corresponding treatment. As the key interest of our study was to characterize individuals with disordered gambling behaviour who access different types of outpatient physician care and not to contrast distinct care pathways, delineating group differences between those patients receiving (differently intense) psychotherapists or neuro-psychiatrists care is beyond the scope of this paper. Thus, it remains to which extent between group heterogeneity is driven by unobserved within group heterogeneity.

Besides these limitations, by using a full census of SHI-insured adults, this study shows the real-world relevance of outpatient psychotherapeutic treatment in addressing GD. As the density of PTs’ offices in Bavaria resembles that of Germany as a whole (67), our findings are most probably generalizable on a national level. Here the broader scope of this study — investigating patients with disordered gambling behavior, both with and without psychotherapeutic treatment — comprehensively captures trends in outpatient treatment for patients with disordered gambling behavior and shows the key role of general practitioners and neuropsychiatrists in the treatment process. In this regard, looking at patient-individual follow-up periods instead of calendar years illustrates annual help-seeking patterns more realistically because date-based censoring is avoided.

## 5 Conclusion

Only a minority of patients with disordered gambling behavior seek outpatient psychotherapeutic treatment and these help-seekers present with a marked mental health comorbidity burden. Therefore, integrated treatment approaches that address both GD and comorbid mental health disorders are highly appropriate. Given the beneficial impact of CBT on GD and the fact that almost all SHI-insured patients with disordered gambling behavior see their general practitioner, sensitizing general practitioners for diagnosing GD and initiating follow-up psychotherapeutic treatment appears to be a promising approach to improve treatment rates. In this regard, a close look at the signs of GD in females is considered worthwhile, as the treatment gap is more pronounced in females.

## Data Availability

The data analyzed in this study is subject to the following licenses/restrictions: Restrictions apply to the availability of these data, which were used within the framework of the contractual agreement. The data are not publicly available due to data protection regulations, but may be obtained from BASHIP upon reasonable request. Requests to access these datasets should be directed to Bavarian Association of Statutory Health Insurance Physicians (BASHIP), Kassenärztliche Vereinigung Bayerns, Elsenheimer Straße 39, 80687 München, Germany, versorgungsforschung@kvb.de.
