# Supplementary material for: Outpatient psychotherapeutic treatment of gambling disorder — lessons learned from Bavaria
Source: Front Psychiatry. 2026 Feb 18;17:1760669. doi: 10.3389/fpsyt.2026.1760669 (PMC12957264; doi:10.3389/fpsyt.2026.1760669)
Supplement: Supplementary file 1 [file Table1.pdf]

Supplementary Table 1: List of billing chiffres

	Billing code
(Psychotherapeutic) Conversation (individual treatment) from chapter 21/22/23	GOP 21220, 22220, 23220
Psychosomatics (individual treatment)	GOP 22221
Hypnosis	GOP 35120
Group psychotherapy primary care	GOP 35173-35179
Differential diagnostic clarification / Verbal intervention in psychosomatic illness (chapter 35 psychosomatic)	GOP 35100, 35110
practicing interventions, (individual treatment)	GOP 35111
Psychotherapeutic Consultation-hour	GOP 35151
Psychotherapeutic acute treatment	GOP 35152
Probationary session (including group treatment)	GOP 35150, GOP 35163-35169, GOP 35173-35179
Analytic psychotherapy (individual treatment)	GOP 35411, 35412, 35415
Depth psychological psychotherapy (individual treatment)	GOP 35401, 35402. 35405
Behavioral therapy (individual treatment)	GOP 35421, 35422, 35425
Behavioral therapy + analytic psychotherapy + depth psychological psychotherapy (group treatment)	GOP 35503-35509, GOP 35513-35519, GOP 35523-35529, GOP 35533-35539, GOP 35543-35549, GOP 35553-35559
